# Construction and validation of a method for automated time label segmentation of heart sounds

**DOI:** 10.3389/frai.2023.1309750

**Published:** 2024-01-11

**Authors:** Liuying Li, Min Huang, Ling Dao, Xixi Feng, Yifeng Liu, Changyou Wei, Fangfang Liu, Jing Zhang, Fan Xu

**Affiliations:** ^1^Department of Traditional Chinese Medicine, Zigong First People's Hospital, Zigong, Sichuan, China; ^2^Department of Physiology, School of Basic Medicine, Chengdu Medical College, Sichuan, China; ^3^Department of Cardiology, The First Affiliated Hospital of Zhengzhou University, Zhengzhou, China; ^4^Department of Clinical Medicine, Chengdu Medical College, Sichuan, China; ^5^Department of Public Health, Chengdu Medical College, Sichuan, China; ^6^Art College, Southwest Minzu University, Sichuan, China; ^7^MOEMIL Laboratory, School of Optoelectronic Information, University of Electronic Science and Technology of China, Chengdu, China

**Keywords:** heart sounds, segmentation, artificial intelligence, audio data analysis tool, justified

## Abstract

Heart sound detection technology plays an important role in the prediction of cardiovascular disease, but the most significant heart sounds are fleeting and may be imperceptible. Hence, obtaining heart sound information in an efficient and accurate manner will be helpful for the prediction and diagnosis of heart disease. To obtain heart sound information, we designed an audio data analysis tool to segment the heart sounds from single heart cycle, and validated the heart rate using a finger oxygen meter. The results from our validated technique could be used to realize heart sound segmentation. Our robust algorithmic platform was able to segment the heart sounds, which could then be compared in terms of their difference from the background. A combination of an electronic stethoscope and artificial intelligence technology was used for the digital collection of heart sounds and the intelligent identification of the first (S1) and second (S2) heart sounds. Our approach can provide an objective basis for the auscultation of heart sounds and visual display of heart sounds and murmurs.

## Introduction

Heart sound signals contain valuable information on the physiology or pathophysiological status of the heart. For example, pathological murmurs can be heard in children with congenital heart disease (Wang J. et al., 2020), and a heart murmur and/or additional heart sounds may indicate a valve sexual heart disease (Long et al., 2020). Cardiac auscultation is currently considered to be the most reliable test for detecting cardiac abnormalities (Esquembre Menor and Castel Sánchez, 1988). For instance, an analysis of the heart sounds recorded using a left ventricular assist device can enable real-time, remote monitoring of the device and cardiac function, for early detection of the left ventricular assist device (LVAD) complications (Chen et al., 2021). A third heart sound (S3) on auscultation may be associated with cardiac insufficiency (Shah and Michaels, 2006), and in heart failure patients, this third heart sound has been independently associated with adverse outcomes, including the progression of heart failure (Drazner et al., 2001).

However, cardiac auscultation requires a qualified cardiologist, and relies on the sounds of the heart cycle (myocardial contraction and valve closure) for the detection of cardiac abnormalities during the pumping action (Boulares et al., 2021). These important heart sounds include the first heart sound (S1), the second heart sound (S2), the third heart sound (S3) and the fourth heart sound (S4). Heart murmurs are short-lived and difficult to capture via human hearing, which may lead to these fleeting physiological signals being ignored. There is therefore an urgent need to find better methods of heart sound analysis.

Recently, the significant growth of artificial intelligence (AI) in the biomedical field has demonstrated elevated levels of accuracy and sensitivity (Rodrigues et al., 2017; Gadaleta et al., 2019; Shen et al., 2021). When it comes to heart sound segmentation, some algorithms have shown strong automated functions. Authors presented a new technique to detect and categorize heart sounds using the self-similarity matrix (SSM) (Rodrigues et al., 2022) for retrieving data from multimodal time series. Their focus is on heart sound segmentation and future potential for automated labeling. Kui et al. (2020) proposed using a combination of the duration hidden Markov model (DHMM) and the Viterbi algorithm to evaluate the localization, feature extraction, and classification of heart sounds in an objective manner. To achieve segmentation results compatible with the sequential nature of heart sounds, authors used the Python package TSSEARCH (Folgado et al., 2022), which is based on the combination of subsequence search and time series similarity measurement with sequential time model. An image-based technology study (Delampady, 2021), inspired by a network utilized for image segmentation, was developed to identify the captured Joint Photographic Experts Group (JPEG) image pattern of the cardiac signal and classify using the hamming distance technique. The segmentation algorithms (Delampady, 2021) incorporated empirical wavelet transform and instantaneous techniques to distinguish heart sounds from murmurs and suppress background noise. Other techniques applied include Shannon entropy envelope extraction, instantaneous phase-based boundary determination, parameter extraction for heart sounds and murmurs, systole/diastole discrimination, and decision-rules based murmur classification. Specifically, measuring the starting point, peak point, and end point of the heart sound signal requires the robust algorithmic capabilities of artificial intelligence (AI).

From the perspective of physical characteristics of the heart sounds, the two dominant variables are the intensity and frequency, as these represent the essential information of the sound (El-Segaier et al., 2005). In addition, an electronic stethoscope was used to record the heart sounds digitally, thus providing a solid foundation for raw data analysis. Here, we introduce a method of automatically segmenting the heart sounds and extracting the fundamental frequency and intensity parameters in combination between audacity and customized MATLAB code. Our method represents a step forward in the artificial intelligence (AI) analysis of heart sounds, and is conducive to the diagnosis and prediction of heart disease.

## Methods

### Ethics statement

The study was approved by the Institute of Institutional Review Board and by Ethics Committee of the Chengdu Medical College. All participants gave informed consent in writing.

### General workflow

**Figure 1:**
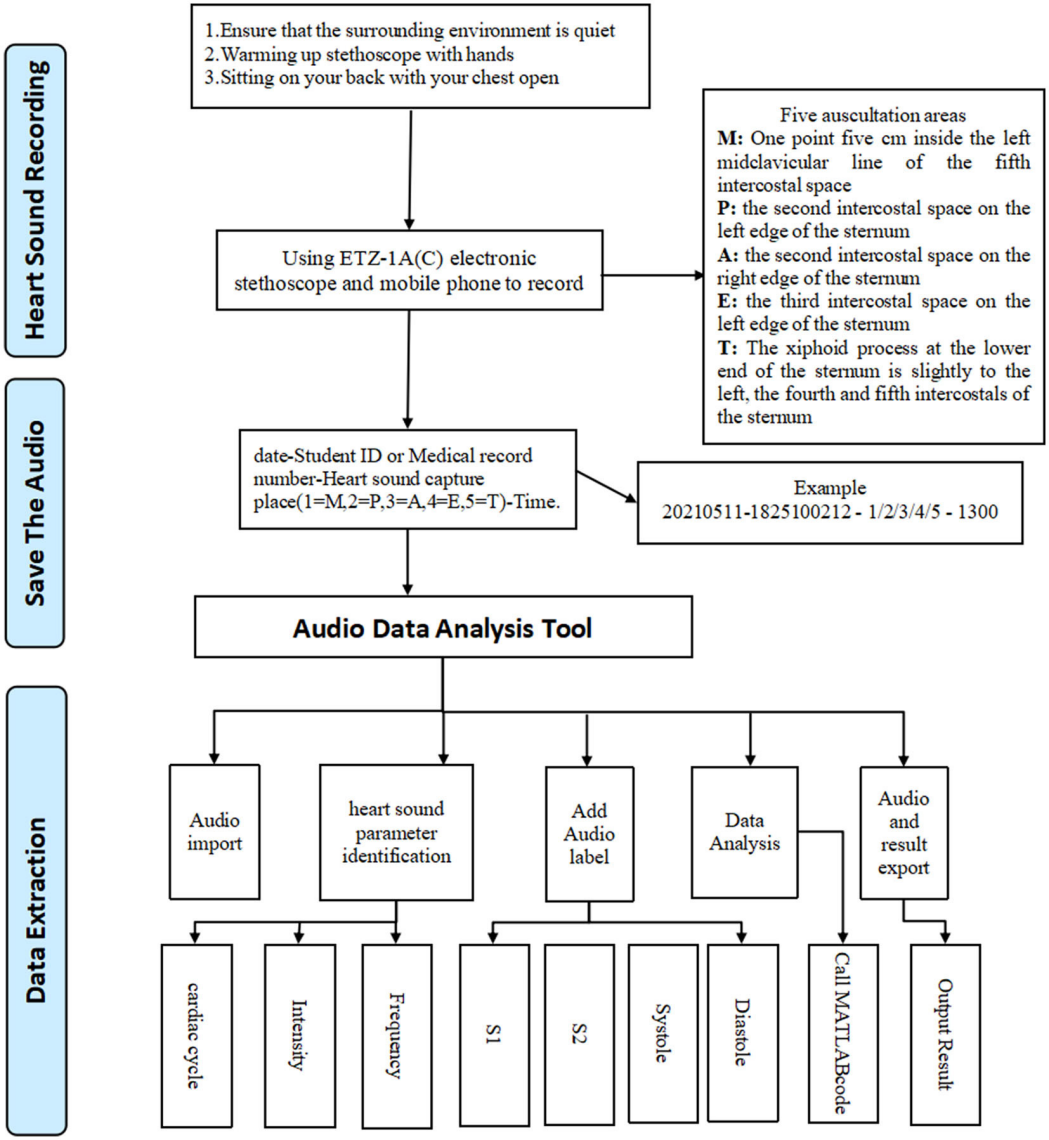
Workflow.

First, we connected the electronic stethoscope and turned on the Moving Picture Experts Group Audio Layer III (mp3) recorder, while keeping the environment quiet. The candidate was asked to sit or take up a supine position with chest exposure. Next, a recorder was placed at the auscultation head with appropriate pressure on each of the five valve areas in turn: the mitral valve area (M), pulmonary valve area (P), first aortic auscultation area (A), second aortic auscultation area (E), and the tricuspid valve area (T), to record heart sounds for a period of at least 1 min. Data from each candidate were collected on three consecutive days, twice a day (8:00–11:00, 14:30–17:00), and 30 audio files were created for each person. Finally, we imported the Moving Picture Experts Group Audio Layer III (mp3) files into our Audio Data Analysis Tool, and automatically cut the audio data representing a cycle into four audio clips (S1, S2, systole, diastole). We then extracted the intensity and frequency parameters as shown in Figure 1.

### Inclusion and exclusion criteria

Healthy adults from Chengdu Medical College with no underlying chronic disease were included. Long-term users of drugs that may cause arrhythmia were excluded. The exclusion criteria were unwilling to continue receiving heart sound collection during the study period.

### Heart sound segmentation

To ensure that the starting point in the audio file was the first heart sound, and to eliminate interference from breathing and external noise, Audacity (version 1.3.3) software was used to display the phonocardiogram. As part of the heart sound analysis, the specific characteristics of the phonocardiogram needed to be analyzed further in terms of the time domain, frequency domain, and loudness. From the results, we searched for the most regular, characteristic heart sound signals for segmentation.

### Splitting the first heart sound (S1) and the second heart sound (S2)

First, our Audio Analysis Data Tool automatically screened the amplitude and time axis of the S1 vertex, according to the baseline at the end of preprocessing, and calculated the frequency range of the S1 peak and the previous wave of the valley. In this way, the start and end times of S1 could be located. Next, we extracted the S2 peak with the second peak between two adjacent S1 peaks as the features and filtered out the S2 peak, which showed a large difference from the average value. We then selected the data with the highest statistical significance and used the times corresponding to the data as labels to obtain the start and end points of S2. Finally, using the statistical data, the start and ending times, state, decibels, amplitude, and other information were calculated for each state. The systolic period, the diastolic period, and the starting time were calculated for S1 and S2.

### Cutting the systolic and diastole segments

One cardiac cycle included a systolic period and a diastolic period, where the systolic period was defined as the time from the start of the first heart sound (S1) to the start of the second heart sound (S2), and the diastolic period was defined as the start of the second heart sound (S2) to the start of the first heart sound (S1) in the next cardiac cycle. The relevant audio clips were cut, and we divided the audio files into separate pieces. By running a customized MATLAB code, the relevant parameters of the systolic and diastolic periods could be extracted.

### Automatic segment coding

Our audio data analysis tool automatically called audacity, using the time labels, to carry out segmentation of the audio files. The four segments were automatically imported into a MATLAB code to calculate the audio, and finally a summary of the data was output.

## Results

### Demographic dataset

We collected data from healthy adults from Chengdu Medical College between 11th May and 6th July 2022. We obtained informed consent from all participants before the experiment. A total of 12 students were recruited, aged between 20 and 22. Demographic information is given in Table 1.

**Table 1:** Demographics of subjects.

**Characteristic**	**Male (mean ±std)**	**Female (mean ±std)**
Number	8	4
Heart rate	72 ± 0.3	73 ± 0.7
Blood oxygen	96.37 ± 0.97	98.17 ± 0.71
Age	20.74 ± 0.43	20.65 ± 0.47
Height	175.48 ± 7.32	162.73 ± 2.69
Weight	65.88 ± 8.58	50.19 ± 2.35
BMI	21.36 ± 2.27	18.94 ± 0.52

### Visualization of heart sound waves via audacity software

**Figure 2:**
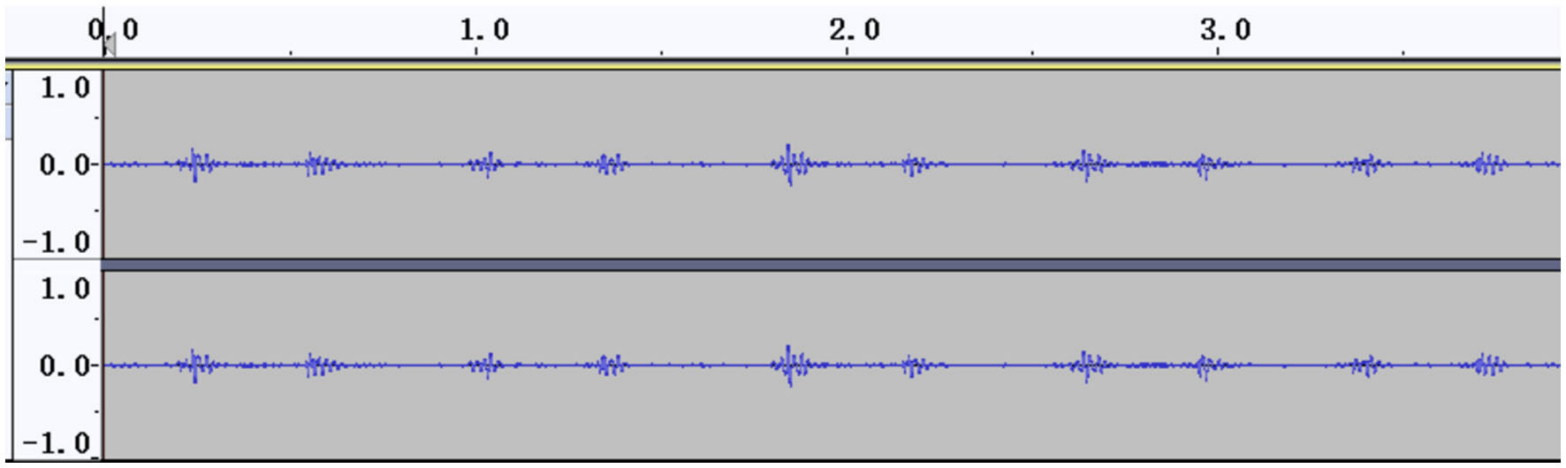
Heart sound cycle before standardization.

**Figure 3:**
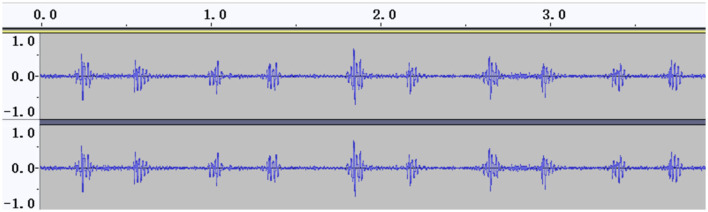
Heart sound cycle after standardization.

The signal amplitudes of the collected heart sounds were small and difficult to identify before standardization (Figure 2). After the standardization process, the amplitude of the heart sound was increased, thus achieving the effect of signal amplification, and the waveform was clearer (Figure 3).

### Cardiac cycle segmentation

**Figure 4:**
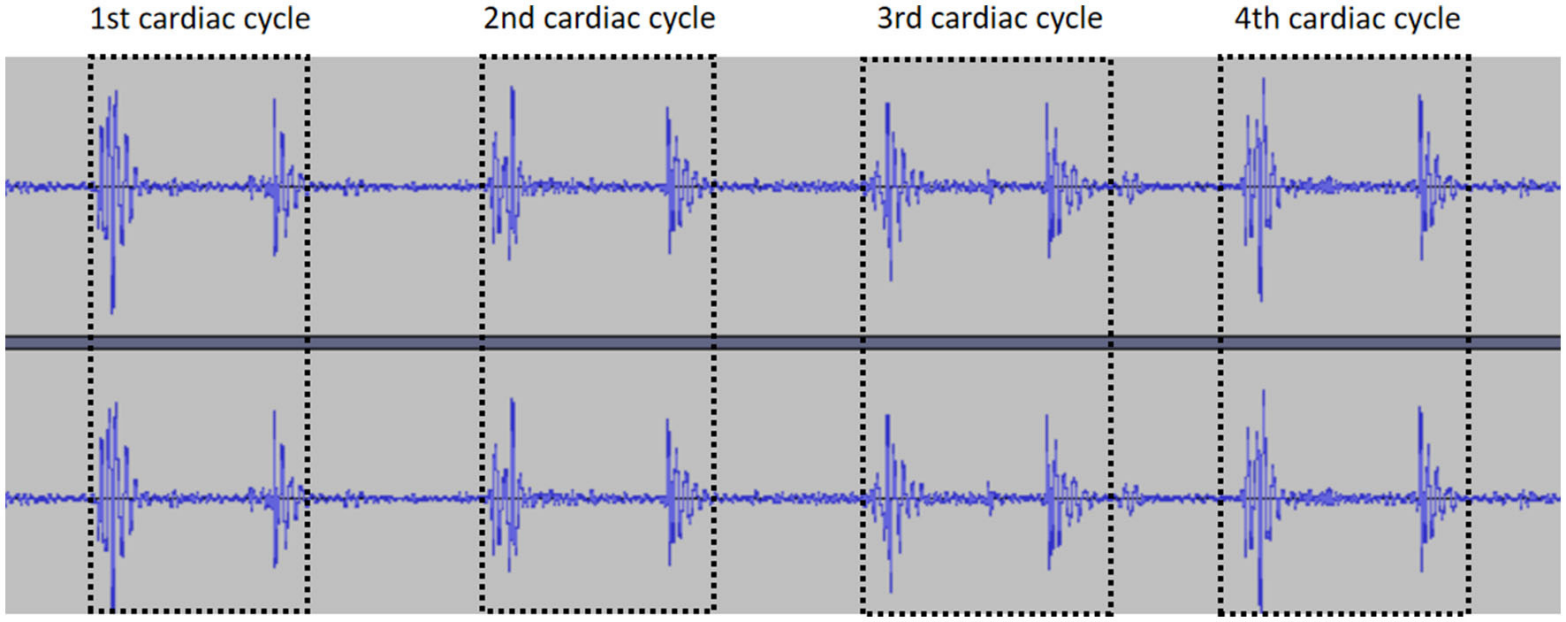
Segmentation of the cardiac cycle.

Segmentation of multiple cardiac cycles from the audio files was carried out based on localization of two adjacent “similar images” (defined as two adjacent images with similar amplitude and duration) in the PCG (Figure 4).

### Splitting S1 and S2 by calculating the start and end positions

**Figure 5:**
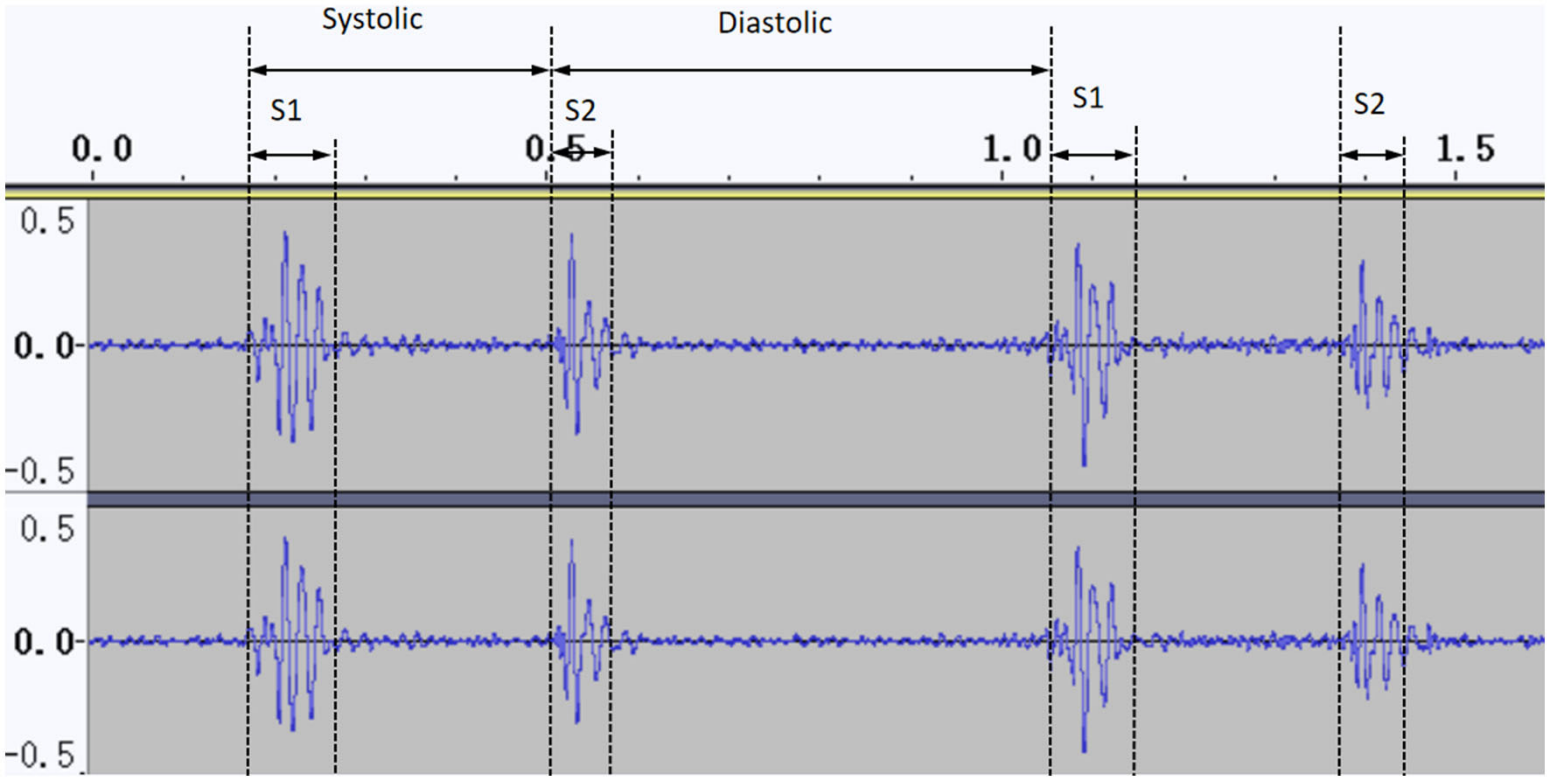
Normal cardiac phonogram signal in audacity.

After locating and segmenting the cardiac cycle, the Audio Data Analysis Tool identified the intensity, time and frequency domain features of S1 and S2 as follows: (i) the duration of S1 is longer than S2; (ii) S1 and S2 have a range of frequencies in physiologically; (iii) S2 occurs between neighboring S1 signals in the PCG (Li et al., 2020). By combining the above features, we identified the start and end positions of S1 and S2 (Figure 5).

### Cutting the systolic and diastole segments based on label information

**Figure 6:**
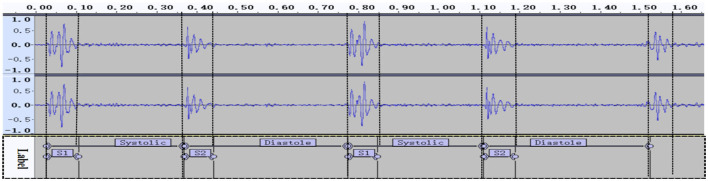
Segment labeling.

To segment the systolic and diastolic phases, we used the time label information to extract them from Audacity. When we had identified and located S1 and S2, we calculated the label value information (Figure 6) and cut the clips accurately.

The recorded heart sounds consisted of the exact start and end time points of the cardiac cycle. These data parameters can be used to represent heart sounds as a set of characteristic signals, based on four parameters that represent and characterize the status of the heart.

### Duration of S1, S2, systolic, and diastolic periods

After obtaining the start and end times of the first and second heart sounds (Figure 6), the duration of the cardiac cycle and the S1, S2, systolic and diastolic periods were calculated (see Table 2).

**Table 2:** Time-domain features.

**Time-domain features**	**Male (mean ±std)**	**Female (mean ±std)**
Cardiac cycle duration	0.83 ± 0.01	0.82 ± 0.01
S1 duration	0.16 ± 0.07	0.12 ± 0.07
S2 duration	0.16 ± 0.07	0.12 ± 0.08
Systolic duration	0.4 ± 0.13	0.42 ± 0.1
Diastolic duration	0.4 ± 0.13	0.42 ± 0.1

### Intensity of maxdb, mindb, meandb, middledb

The amplitudes of the heart sounds can represent the intensity of the mechanical activity of the heart, which may be helpful in the detection of coronary heart disease (Liu et al., 2021). Audacity was used to derive the loudness values, which were calculated and filtered. The amplitude of each segment of the cardiac cycle (S1, S2, systolic, and diastolic) was calculated. For this work, the mean value and SD of the amplitude features (maxdb, mindb, meandb, middledb) for the heart cycle were calculated, as shown in Table 3.

**Table 3:** Amplitude features.

**Amplitude features**	**S1**		**S2**		**Systolic**		**Diastolic**	
	**Male**	**Female**	**Male**	**Female**	**Male**	**Female**	**Male**	**Female**
maxdb	−7.42 ± 5.19	−12.11 ± 7.9	−7.89 ± 5.67	−13.13 ± 7.98	−7.42 ± 5.19	−12.11 ± 7.9	−7.89 ± 5.67	−13.13 ± 7.98
mindb	−6.9 ± 5.28	−11.86 ± 7.68	−7.64 ± 5.81	−12.82 ± 7.78	−6.9 ± 5.28	−11.86 ± 7.68	−7.64 ± 5.81	−12.82 ± 7.78
meandb	−22.87 ± 5.68	−26.59 ± 7.84	−23.6 ± 5.93	−27.17 ± 7.28	−27.74 ± 7.59	−35.36 ± 12.46	−28.09 ± 7.27	−35.64 ± 12.21
middledb	−7.16 ± 5.06	−11.98 ± 7.69	−7.76 ± 5.56	−12.97 ± 7.78	−7.16 ± 5.06	−11.98 ± 7.69	−7.76 ± 5.56	−12.97 ± 7.78

### Frequency

Spectral analysis is the most widely used method of heart sound analysis. In this study, a MATLAB code (see attachment) was used to perform formula calculation for the four segments (S1, S2, systolic, and diastolic). A detailed summary of the results is given in Table 4.

**Table 4:** Frequency features.

**Frequency features**	**S1**		**S2**		**Systolic**		**Diastolic**	
	**Male**	**Female**	**Male**	**Female**	**Male**	**Female**	**Male**	**Female**
absomean	0.15 ± 0.09	0.08 ± 0.09	0.14 ± 0.08	0.07 ± 0.08	0.1 ± 0.08	0.05 ± 0.08	0.1 ± 0.07	0.05 ± 0.08
STD	0.2 ± 0.1	0.1 ± 0.12	0.19 ± 0.1	0.1 ± 0.11	0.15 ± 0.1	0.07 ± 0.11	0.15 ± 0.08	0.07 ± 0.1
skew	−0.15 ± 0.58	−0.12 ± 0.34	−0.07 ± 0.61	−0.12 ± 0.38	−0.16 ± 0.81	−0.16 ± 0.69	−0.04 ± 0.65	−0.2 ± 0.66
kurt	4.41 ± 2.73	3.92 ± 3.8	4.5 ± 2.63	3.68 ± 2.87	9.63 ± 9.83	16.68 ± 15.89	11.04 ± 10.31	21.02 ± 22.68
max	0.5 ± 0.23	0.25 ± 0.25	0.49 ± 0.23	0.23 ± 0.24	0.51 ± 0.23	0.27 ± 0.27	0.61 ± 0.2	0.31 ± 0.27
min	−0.53 ± 0.24	−0.26 ± 0.25	−0.5 ± 0.24	−0.25 ± 0.26	−0.54 ± 0.24	−0.28 ± 0.27	−0.61 ± 0.21	−0.33 ± 0.28
peak2valley	1.02 ± 0.45	0.51 ± 0.49	0.99 ± 0.46	0.48 ± 0.49	1.06 ± 0.45	0.55 ± 0.53	1.22 ± 0.39	0.64 ± 0.54
RMS	0.2 ± 0.1	0.11 ± 0.12	0.19 ± 0.1	0.1 ± 0.11	0.15 ± 0.1	0.07 ± 0.11	0.15 ± 0.08	0.07 ± 0.1
shapefactor	1.38 ± 0.16	1.34 ± 0.23	1.38 ± 0.16	1.31 ± 0.16	1.6 ± 0.36	1.95 ± 0.56	1.67 ± 0.33	2.01 ± 0.53
impulsefactor	3.7 ± 1.44	3.54 ± 2.49	3.89 ± 1.63	3.32 ± 1.7	6.82 ± 4.77	11.46 ± 8.63	8.45 ± 5.1	13.99 ± 11.32
marginfactor	39.55 ± 41.17	90.69 ± 147.94	46.62 ± 56.11	85.99 ± 109.23	189.99 ± 416.86	1044.91 ± 1302.16	217.53 ± 444.88	1295.39 ± 1783.67
energy	135.32 ± 168.91	66.48 ± 180.09	122.21 ± 168.14	59.52 ± 159.03	191.58 ± 271.58	110.73 ± 282.29	378.7 ± 509.87	208.84 ± 518.58
first_f0	369.02 ± 88.66	272.82 ± 157.53	369.57 ± 87.15	308.21 ± 144.55	361.4 ± 97.48	252.38 ± 155.85	361.4 ± 97.48	252.38 ± 155.85
middle_f0	377.78 ± 74.94	299.31 ± 148.98	376.37 ± 77.74	332.88 ± 128.09	373.91 ± 84.53	247.79 ± 155.12	372.87 ± 86.04	267.36 ± 152.82
last_f0	381 ± 71.97	338.86 ± 122.64	377.82 ± 74.14	346.69 ± 116.39	368.7 ± 92.63	253.06 ± 153.8	374.37 ± 83.43	244.78 ± 156.01
median_f0	379.02 ± 73.13	329.71 ± 124.05	376.59 ± 76.89	348.7 ± 106.31	373.34 ± 84.8	277.08 ± 142.09	370.8 ± 88.91	278.61 ± 141.77
mean_f0	377.6 ± 67.65	317.8 ± 108.34	376.04 ± 70.47	340.7 ± 96.41	371.83 ± 75.28	263.03 ± 96.76	371.28 ± 75.27	259.03 ± 91.85
f0variation	13.43 ± 37.54	59.53 ± 81.5	12.43 ± 35.38	46.21 ± 76.21	20.13 ± 40.65	101.15 ± 63.61	24.12 ± 38.98	106.56 ± 61.57
f0skew	−0.71 ± 1.5	−0.48 ± 1.07	−0.83 ± 1.61	−0.42 ± 1.13	−1.82 ± 2.8	−0.34 ± 1.25	−3.4 ± 3.6	−0.25 ± 1.09
f0kurt	3.94 ± 4.88	2.46 ± 3.08	4.41 ± 5.73	2.76 ± 2.46	12.61 ± 13.93	3.06 ± 4.03	26.23 ± 26.98	2.71 ± 3.01
max_f0	389.66 ± 49.55	364.42 ± 92.44	387.22 ± 56.06	379.44 ± 67.86	395.27 ± 30.42	369.31 ± 63.86	399.16 ± 9.82	376.25 ± 51.19
min_f0	356.39 ± 106.15	245.67 ± 166.01	356.77 ± 102.23	285.61 ± 158.46	328.27 ± 130.19	116.78 ± 133.27	296.3 ± 142.47	106.52 ± 125.69
range_f0	33.27 ± 89.61	118.75 ± 156.83	30.45 ± 82.77	93.83 ± 143.62	66.99 ± 123.48	252.53 ± 134.37	102.86 ± 141.36	269.73 ± 125.64
slope_start2max	0.95 ± 0.17	0.78 ± 0.36	0.96 ± 0.16	0.83 ± 0.32	0.91 ± 0.23	0.68 ± 0.37	0.9 ± 0.24	0.66 ± 0.38
slope_max2end	1.1 ± 0.61	1.34 ± 1.21	1.1 ± 0.64	1.45 ± 1.37	1.34 ± 1.19	2.65 ± 2.33	1.3 ± 1.14	2.87 ± 2.42
jitter	0	0	0	0	−13.57 ± 33.07	−2.76 ± 23.05	−12.81 ± 32.45	0.48 ± 5.24
HNR	−6.43 ± 24.27	−1.14 ± 6.62	−7.77 ± 17.66	−2.51 ± 8.14	0	0	0	0
hr_mean	0.07 ± 0.05	0.19 ± 0.08	0.07 ± 0.04	0.19 ± 0.08	0.05 ± 0.03	0.19 ± 0.08	0.05 ± 0.02	0.19 ± 0.08
hr_median	0.03 ± 0.04	0.16 ± 0.09	0.03 ± 0.04	0.16 ± 0.1	0.01	0.17 ± 0.09	0.01	0.17 ± 0.09
hr_std	0.11 ± 0.06	0.12 ± 0.05	0.1 ± 0.05	0.12 ± 0.05	0.1 ± 0.04	0.12 ± 0.03	0.11 ± 0.03	0.12 ± 0.02
hr_max	0.35 ± 0.19	0.42 ± 0.16	0.34 ± 0.19	0.44 ± 0.16	0.45 ± 0.19	0.54 ± 0.14	0.58 ± 0.15	0.64 ± 0.11
hr_min	0.01	0.07 ± 0.06	0.01	0.08 ± 0.07	0	0.04 ± 0.03	0	0.02 ± 0.02

Max, maximum amplitude; min, minimum amplitude; RMS, representation of the stability and reproducibility of the signal; HNR, harmonic noise ratio; STD, standard deviation.

### Cross-validation based on heart rate

To cross-validate the accuracy of our segmentation results, we also used a finger oxygen monitor to record the heart rates of the participants during the data collection process. The details of the measurement results are shown in Table 5. The result of a paired *T*-test indicated that no significant difference was found between the two methods.

**Table 5:** Cross-validation based on heart rate.

**Candidate**	**Results from automated time label segmentation method**	**Results from finger oxygen monitor**
Subject 01	73	73
Subject 02	74	73
Subject 03	73	73
Subject 04	72	72
Subject 05	73	73
Subject 06	72	73
Subject 07	73	73
Subject 08	73	73
Subject 09	73	73
Subject 10	73	73
Subject 11	73	73
Subject 12	71	72
Mean (sd)^*^	72.73 (0.78)	72.82 (0.40)

^*^Paired t-test, t = −1.43, df = 500, P = 0.152.

## Discussion

Cardiovascular disease is one of the most common diseases threatening human health worldwide, while the robust and non-invasive diagnostic techniques are urgently needed. In particular, heart sound detection techniques can play an important role in the prediction of cardiovascular disease. However, traditional cardiac auscultation requires a qualified cardiologist, as heart murmurs are short-lived and difficult to identify via human hearing.

Artificial intelligence recognition technology can detect the duration, intensity, frequency, and other acoustic information of the heart sound signal; this approach also has the advantages of batch processing and convenience in terms of the screening and detection of disease. In this work, we constructed and validated a method of automatically segmenting heart sounds and extracting the fundamental frequency and intensity parameters through our Audio Data Analysis Tool. We also showed that there were no differences in the heart rates found by the Audio Data Analysis Tool and a finger oxygen monitor, see Table 5.

Considerable amounts of work have been directed toward reaching the goal of diagnosis based on the detection of heart sounds recorded from the chest. One clinical trial demonstrated that deep learning algorithms were comparable to cardiologists in their ability to detect murmurs and clinically significant aortic stenosis and mitral regurgitation. An eighth-order Butterworth high-pass filter is used at the input end of the algorithm design. The model comprises a 1-dimensional convolution, batch normalization, rectified linear unit non-linearity, dropout for regularization, and maximum pooling (Chorba et al., 2021). A preliminary study by Ghanayim et al. (2022) found that an AI-based algorithm identified moderate or severe mitral stenosis with 86% sensitivity and 100% specificity. They proposed an AI-based algorithm that tests and validates multiple machine learning methods to determine the best algorithm, integrated into an intelligent stethoscope, was able to accurately diagnose aortostenosis (AS) in seconds. Wang J. K. et al. (2020) presented a neural network model consisting of convolution, recurrence, time-attentive pool (TAP), and dense slices that accurately identifies systolic heart sounds in patients with ventricular septal defects and may be able to classify heart sounds associated with several other structural heart diseases. The study shared similarities with ours, beginning with electronic stethoscope-based data collection and successive signal preprocessing, feature extraction, and final classification stages, resulting in a comprehensive analysis of heart sounds. However, it diverges in its application of machine learning models (Omarov et al., 2023).

A comparison of other methods of extracting heart sound signals shows that our innovation was to use Audacity to carry out time label aggregation of heart sounds, which made it easy to locate problems when outputting data errors. In addition, all of the acoustic parameters could be collected; for example, the data contained five duration indicators, 16 loudness indicators, and 60 frequency indicators. This also makes it convenient for researchers to process data diversification.

In general, the problem of analyzing heart sound signals arises from the substantial amount of noise involved, such as breath sounds, heartbeat rhythms and voices in the external environment. Unfortunately, we could only achieve noise reduction through the proficiency of trained cardiologists, and this will be a direction for future work. In addition, at the preprocessing stage, our process involved cutting at the beginning and ending of the audio files, which may lead to a little deviation.

## Conclusion

A combination of an electronic stethoscope and artificial intelligence technology was applied to the digital collection of heart sounds and the intelligent identification of the first (S1) and second (S2) heart sounds. Our approach provides an objective basis for the auscultation of heart sounds and visual display of heart sounds and murmurs.

## Data availability statement

The raw data supporting the conclusions of this article will be made available by the authors, without undue reservation.

## Ethics statement

The studies involving humans were approved by the Zigong First People's Hospital. The studies were conducted in accordance with the local legislation and institutional requirements. Written informed consent for participation in this study was provided by the participants' legal guardians/next of kin.

## Author contributions

LL: Investigation, Resources, Writing—original draft. MH: Data curation, Methodology, Validation, Visualization, Writing—review & editing. LD: Data curation, Formal analysis, Resources, Validation, Visualization, Writing—review & editing. XF: Data curation, Writing—original draft. YL: Formal analysis, Funding acquisition, Project administration, Resources, Writing—review & editing. CW: Methodology, Project administration, Writing—review & editing. FL: Conceptualization, Visualization, Writing—review & editing. JZ: Writing—review & editing. FX: Conceptualization, Formal analysis, Funding acquisition, Writing—original draft, Writing—review & editing.
